# Alpha5 nicotinic acetylcholine receptor mediated immune escape of lung adenocarcinoma via STAT3/Jab1-PD-L1 signalling

**DOI:** 10.1186/s12964-022-00934-z

**Published:** 2022-08-15

**Authors:** Ping Zhu, Zhengxin Jin, Guiyu Kang, Yanfei Jia, Duanrui Liu, Qian Zhang, Feiyang Guo, Ying Jia, Yang Jiao, Jingtan Li, Haiji Sun, Xiaoli Ma

**Affiliations:** 1 Research Center of Basic Medicine, Central Hospital Affiliated to Shandong First Medical University, Jinan, China; 2 Department of Medical Laboratory, Weifang Medical University, Weifang, China; 3 Research Center of Basic Medicine, Jinan Central Hospital, Shandong University, Jinan, China; 4 College of Life Science, Shandong Normal University, Jinan, China

**Keywords:** α5-nAChR, STAT3/Jab1-PD-L1, Immune escape, Lung adenocarcinoma

## Abstract

**Background:**

Immunotherapy has proven to be an emerging treatment for non-small-cell lung cancer in recent years. Notably, smokers show higher programmed cell death ligand-1 (PD-L1) expression and better responses to PD-1/PD-L1 inhibitors than nonsmokers. Genome-wide association studies show that the *CHRNΑ5* encoding α5-nicotinic acetylcholine receptor (α5-nAChR) is especially relevant to lung cancer and nicotine dependence. Jab1 is a key regulatory factor and promotes the stabilization of PD-L1. Our previous study reported that α5-nAChR mediates lung adenocarcinoma (LUAD) epithelial-mesenchymal transition (EMT) and metastasis via STAT3/Jab1. However, the link between α5-nAChR and PD-L1 is unclear in LUAD.

**Methods:**

We used various bioinformatics databases to analyze the expression of related genes and their correlations. Expression and clinicopathologic significance of α5-nAChR and PD-L1 were detected by immunohistochemistry in a tissue microarray. α5-nAChR regulated LUAD cell immune escape by targeting the STAT3/Jab1-PD-L1 signalling by Western-blotting and ChIP in vitro. We used T cell coculture, flow cytometry, ELISA, CCK8 assay and crystal violet staining to detect the expression of regulatory T cell (Tregs), IFN-γ, IL-2 and the ability of T cell-mediated tumour cell killing respectively. IF assays were performed in both cancer cells and tumour xenograft paraffin sections to analyze the protein expression. The in vivo experiments in mouse model were performed to show the α5-nAChR-mediated immune escape via PD-L1 pathway.

**Results:**

The expression of α5-nAChR was correlated with PD-L1 expression, smoking status and lower survival of LUAD in vivo. In vitro, the expression of α5-nAChR mediated phosphorylated STAT3 (pSTAT3), Jab1 and PD-L1 expression. STAT3 bound to the Jab1 or PD-L1 promoter and mediated PD-L1 expression. Jab1 stabilized PD-L1 expression in LUAD cells. Furthermore, in primary T cell cocultured system, downregulation of α5-nAChR suppressed the function of CD4^+^CD25^+^FOXP3^+^ Tregs, enhanced IFN-γ secretion, and increased T cell-mediated killing of LUAD cells. In the Jurkat T cells and LUAD cells coculture assay, inhibition of α5-nAChR increased IL-2 secretion. In tumour xenograft tissues, α5-nAChR expression was related to PD-L1, Jab1, pSTAT3, CD4 and granzyme B expression (GB).

**Conclusions:**

Our results suggest that the novel α5-nAChR/STAT3-Jab1-PD-L1 axis is involved in LUAD immune escape, which could lead to potential therapeutic strategies for cancer immunotherapy.

**Video abstract**

**Supplementary Information:**

The online version contains supplementary material available at 10.1186/s12964-022-00934-z.

## Introduction

Lung cancer is one of the malignant tumours with the highest incidence and mortality worldwide. NSCLC is the most common tissue type in lung cancer, accounting for 80–85% of all types of lung cancer [1]. In the past decade, advances in epidermal growth factor receptor (EGFR) therapy [2] and anaplastic lymphoma kinase (ALK) therapy [3] have remarkably improved the survival of EGFR positive and ALK positive lung cancer patients. However, effective therapeutic strategies for NSCLC are still desperately needed.

Immunotherapy has proven to be a promising treatment for NSCLC in recent years. PD-L1 is a type 1 transmembrane protein that is encoded by the *CD274* gene in humans and is overexpressed in some kinds of cancers [4]. Programmed cell death protein 1 (PD-1) is an inhibitory receptor that is encoded by the *PDCD1* gene and is located on the surface of T cells [5]. The PD-1/PD-L1 axis generates an inhibitory signal that attenuates the activity of T cells and contributes to tumour immune escape [6, 7]. NSCLCs have higher expression of PD-L1, which is an immune checkpoint molecule and an important target in immunotherapy[8]. In lung cancer, high PD-L1 expression is associated with larger tumour size, higher tumour grade, and lymph node number. According to the outcomes in clinical trials, smoking is associated with a significantly better anti-PD-1 monotherapy response [9]. Several recent studies have shown positive correlations between smoking and PD-L1 expression or PD-1/PD-L1 immune checkpoint inhibitor efficacy and better treatment responses to anti-PD-1/PD-L1 immunotherapy than patients with lung cancer who have never smoked [10].

Nicotinic acetylcholine receptors (nAChRs) are ligand-gated ion channel proteins comprising pentameric subunits using various combinations of alpha (α1-α10) or non-α (β1–β4, γ, δ, or ε) type openings and are regulated by the binding of acetylcholine or nicotine agonists, such as nicotine [11]. nAChRs are classified into two categories, heteromeric and homomeric nAChRs. Homomeric nAChRs are composed of five α subunits (α7, α8, and α9), and other subunits combine to create a variety of receptors with different functional and pharmacological properties[12]. α5-nAChR is one of the most recently discovered nAChR subtypes and is encoded by the *CHRNΑ5* gene in humans [13–15]. GWAS have shown that α5-nAChR is highly associated with lung cancer risk and nicotine dependence [16, 17].Our previous study reported that α5-nAChR mediates nicotine-induced LUAD development and progression via the PI3K and JAK2/STAT3 pathways [18–20]. Nevertheless, the link between α5-nAChR and PD-L1 is unclear in LUAD.

Jab1 has shown to be the fifth member of the COP9 signalosome complex, which is encoded by the *COPS5* gene [21]. Recent studies have shown that Jab1 is a potential target for smoking-induced lung cancer that mediates various tumorigenesis-associated pathways [22, 23]. Jab1 inhibits the ubiquitination and degradation of PD-L1 [24, 25]. Downregulation of Jab1 decreases PD-L1 expression in cancer cells. Notably, our previous study demonstrated that α5-nAChR mediates LUAD cells EMT, migration and invasion via STAT3/Jab1 signalling [26]. Is α5-nAChR associated with PD-L1 via STAT3/Jab1 in lung cancer immune escape?

In this study, we assessed α5-nAChR and PD-L1 expression in LUAD in vivo and demonstrated that α5-nAChR expression was correlated with PD-L1 expression, smoking status and lower survival. α5-nAChR mediated immune escape via STAT3/Jab1-PD-L1 signalling in NSCLC cells. To our knowledge, this is the first study to show that the α5-nAChR/STAT3-Jab1-PD-L1 axis is involved in immune escape in lung cancer.

## Methods

### *CHRNA5* and *CD274* expression and clinical characteristics using relevant databases

The KM-plotter online analysis tool (http://kmplot.com/analysis) was used to analyze the overall survival (OS) of lung cancer patients. We downloaded α5-nAChR and PD-L1 gene expression data from the cancer genomic browser of UCSC Xena (https://xenabrowser.net/) to test the prognostic value of high coexpression of *CHRNA5* and *CD274* in LUAD. Primary tumor samples were categorized using clinical patient data and the UALCAN database (http://ualcan.path.uab.edu/index.html) was used to generate boxplots of *CHRNA5* and *CD274* expression levels from LUAD smokers and nonsmokers. We used the R2 online database (http://r2.amc.nl) and selected various microarray datasets to analyze the correlations among *CHRNA5*, *STAT3 CD274*, and granzyme B gene (*GZMB*). The mRNA results of *CHRNA5* were analyzed using GSEA software. A *P* value < 0.05 was considered statistically significant.

### Tissue specimens and cell culture

A tissue microarray (No. OD-CT-RsLug04-003, OUTDO BIOTECH) containing 55 lung adenocarcinoma specimens and 53 paracarcinoma tissues with smoking status was used in this study. Each set of paired tumour and paracarcinoma tissues was collected and categorized according to their clinical information. Among the 55 samples, there were 30 males and 25 females with an age range of 45–82 years (age average, 64.2 years).

All experiments were performed with mycoplasma-free cells. The human LUAD cell lines Α549 and H1299 were purchased from the Cell Resource Center of the Chinese Academy of Sciences. Jurkat T cells were purchased from Shanghai Zhong Qiao Xin Zhou Biotechnology Co., Ltd. LUAD cell lines, primary T cells and Jurkat T cells were grown in RIPM 1640 medium (HyClone) supplemented with 10% foetal bovine serum (Gibco) and 1% penicillin and streptomycin (Macgene). Cells were cultured in a humidified atmosphere of 5% CO2 at 37 °C. Human LUAD cell lines were treated with 1 µM nicotine (Sigma) for 16 h as described in our previous study [18]. Cycloheximide (CHX) was purchased from Sigma–Aldrich. MG-132 was obtained from MedChemExpress. BMS-1(PD-1/PD-L1 inhibitor 1) was purchased from Selleck. All human cell lines have been validated within the past three years using STR analysis.

### Xenograft tumour model

The animal assays were approved by the Institutional Animal Care and Use Committee of Central Hospital Affiliated to Shandong First Medical University. Male C57BL/6 mice and BALB/c nude mice (18–22 g, 4–6 weeks old) were purchased from Beijing Vital River Laboratory Animal Technology Co., Ltd and used for in vivo studies. Paraffin sections of BALB/c nude mice lung tumour xenografts were obtained from our laboratory [19].

In C57BL/6 mice, 5 × 10^7^ cells stably overexpressing α5-nAChR (α5-nAChR-OE) or control cells (NC) suspended in 100 μL of medium were subcutaneously injected into the mice. Tumours were evaluated every 2 days, and the volume was calculated by the following formula: length × width^2^/2. When the tumour volume reached approximately 50 to 70 mm^3^, 10 mg/kg BMS-1 was administered via intraperitoneal injection every 2 days in NC group (NC + BMS-1) and α5-nAChR-OE group (α5-nAChR-OE + BMS-1). Mice were sacrificed after 8 times treatment of the BMS-1, tumour tissues were collected for analyses.

### Immunohistochemistry

Tissue sections (4 µm thickness) were detected by immunohistochemical staining using the streptavidin peroxidase method. Paraffin sections were baked at 65 °C for 1.5 h, deparaffinized in xylene, rehydrated through graded ethanol, processed for antigen retrieval in citrate antigen retrieval solution (pH = 6.0) or EDTA buffer (pH = 9.0) for approximately 10 min, and quenched for endogenous peroxidase activity in 3% hydrogen peroxide for 10 min. Sections were blocked with normal serum at room temperature for 2 h. The samples on the slides were incubated at 4 °C overnight with a mouse monoclonal antibody against α5-nAChR (1:200, Proteintech, Cat. No. 66363-1-Ig), a mouse monoclonal antibody against PD-L1 (1:500, Proteintech, Cat. No. 66248-1-Ig), a rabbit monoclonal antibody against pSTAT3 (1:200, CST, Cat. No. 9145), a rabbit polyclonal antibody against Jab1 (1:500, Proteintech, Cat. No. 27511-1-AP), a rabbit monoclonal antibody against CD4 (1:1000, Abcam, Cat. No. ab183685), a rabbit monoclonal antibody against GB (1:3000, Abcam, Cat. No. ab255598). Tissue sections were incubated with an enzyme‐enhanced goat anti‐mouse or rabbit IgG polymer for 20 min at room temperature.

The staining scores were evaluated independently by two investigators, who were blinded to the information on the microarrays. For IHC analysis, the grading was as follows: undyed was scored as 0, yellow was scored as 1, and brown was scored as 2. A percentage of positive tumour cells in the visual field < 1% was scored as 0, while 1–25% was scored as 1, 25–75% was scored as 2, and 75–100% was scored as 3.

### Small interfering RNA transfection and lentiviral infection

The siRNAs for *CHRNA5* and *COPS5* and the negative control were obtained from RiboBio. A549 or H1299 cells were plated in six-well plates. When cells reached 60–70% confluence, the siRNAs were added to a final concentration of 15 nM with Lipofectamine 2000 (Invitrogen) according to the manufacturer's instructions. The sequences of small interfering RNA were as follows: si-CHRNA5 5′-GGTCCGCAAGATATTTCTT-3′, si-COPS5 5′-GTACCAGACTATTCCACTT-3′, the siRNA sequences of STAT3 as described previously [20].

Human CHRNA5 cDNA was subcloned into a pGV-puro lentiviral vector containing the puromycin resistance to establish stable A549 cell line to induce α5-nAChR overexpression (marked as α5-nAChR-OE). The infected cells were selected by puromycin for at least 1 week.

### Western blotting

Cells were harvested, and proteins were extracted with RIPA lysis buffer and a Nuclear and Cytoplasmic Protein Extraction Kit (Beyotime, China). For Western blotting analysis, protein was separated on a 10% SDS–PAGE gel and transferred onto nitrocellulose membranes (0.45 μm, Millipore, USA), which were then incubated at 4 °C overnight. The following antibodies were used: α5-nAChR (1:800, GeneTex, Cat. No. GTX55490), pSTAT3 (1:2000, CST, Cat. No. 9145), STAT3 (1:2000, CST, Cat. No. 4904), Jab1 (1:2000, Proteintech, Cat. No. 27511-1-AP), PD-L1 (1:5000, Proteintech, Cat. No. 66248-1-Ig), Lamin B (1:2000, Proteintech, Cat. No. 12987-1-AP) and GAPDH (1:10,000, Proteintech, 10494-1-AP). Blots were then incubated in horseradish peroxide-conjugated anti-rabbit IgG antibody (1:10,000) and anti-mouse IgG antibody (1:10,000).

### Chromatin immunoprecipitation (ChIP)

The chromatin immunoprecipitation assay was conducted using SimpleChIP^@^ Plus Kits Reagents (9004S, CST) following the manufacturer’s instructions. Briefly, cells were cultured to approximately 1 × 10^7^ and cross-linked with 1% formaldehyde. Samples were then harvested, and chromatin was digested with micrococcal nuclease. DNA fragment length was breaked to be 150–900 bp. Chromatin was immunoprecipitated with either control IgG or STAT3 (1:2000, CST, Cat. No. 4904) primary antibody. Precipitated DNA was amplified by real-time PCR using primers targeting the PD-L1 or Jab1 promoter that have been shown to interact directly with STAT3 [27, 28]. The primer sequences were as follows: PD-L1 forward 5′-GACCTGGCTGCACTAATTGTC-3′ and reverse 5′-CATTTCCCAGGGAG AGCTGG-3′; Jab1 forward 5′-CCTCTCAACC AGCTTCCAGAACG-3′ and reverse 5′-TGTGGAGGGCGGAAAGTGTT-3′. Nonimmunoprecipitated chromatin fragments were used as an input control.

### Primary T cell coculture, flow cytometry and IFN-γ production assay

Whole blood was obtained from healthy donors with informed consent and approved by the Ethics Committee of Central Hospital Affiliated to Shandong First Medical University (JNCH2021-77). Peripheral blood mononuclear cells (PBMCs) were purified using Human Lymphocyte Separation Medium (DAKEWE, China). Briefly, the whole blood diluted with PBS was spread on the separation solution. After centrifugation, the specific gravity of lymphocytes and monocytes were less than or equal to the specific gravity of the separation medium, floating on the surface of the separation medium. Mononuclear cells could be isolated from peripheral blood by absorbing cells on the surface of the separation fluid. PBMCs were stimulated with 2 μg/mL anti-CD3 (BioLegend, Cat. No. 317326) and 2 μg/mL anti-CD28 (BioLegend, Cat. No. 302934) in medium containing 200 U/mL human interleukin-2 (IL-2, PeproTech, Cat. No. 200–02) for 3 days.

A total of 1 × 10^5^ A549 or H1299 cells were seeded in 12-well plates. After the cells adhered and were transfected with si-NC or si-CHRNA5, the supernatants were discarded. Primary T cells were added to A549 or H1299 cells at a ratio of 5:1 in 1 ml media. Afterwards, primary T cells were incubated with CD4 antibody (1:20, BioLegend, Cat. No. 317408) and CD25 antibody (1:20, BioLegend, Cat. No. 302610) for 30 min. Samples were then fixed and permeabilized with True-Nuclear™ Transcription Factor Buffer Set (BioLegend, Cat. No. 424401) according to the manufacturer’s instructions. Samples were then incubated with FOXP3 antibody (1:20, BioLegend, Cat. No. 320008) for 60 min and analyzed by flow cytometry. The supernatants were collected and examined by a Human IFN-γ Precoated ELISA kit (DAKEWE, China) according to the manufacturer’s directions.

### Jurkat T cells cocultured with tumor cells and ELISA assay

Jurkat T cells were stimulated with 2 μg/mL anti-CD3 (BioLegend, Cat. No. 317326) and 2 μg/mL anti-CD28 (BioLegend, Cat. No. 302934) in medium containing 200 U/mL human interleukin-2 (IL-2, PeproTech, Cat. No. 200–02) for 3 days. A total of 1 × 10^5^ A549 or H1299 cells were seeded in 12-well plates. After the cells adhered and were transfected with si-NC or si-CHRNA5, the supernatants were discarded. Jurkat T cells were added to A549 or H1299 cells at a ratio of 5:1 in 1 ml media. The supernatants were collected after 24 h and examined by a Human IL-2 Precoated ELISA kit (DAKEWE, China) according to the manufacturer’s instructions.

### T cell-mediated tumour cell killing assay

We cocultured tumour cells with activated primary human T cells to analyze the killing of tumour cells by T cells and determined the killing by CCK-8 assay or crystal violet staining. Briefly, A549 and H1299 cells were cocultured with activated T cells for 24 h. CCK-8 solution (10 μL) was added to each well and incubated for 2.5 h. Cell viability was analyzed by a microplate reader at 450 nm.

For crystal violet staining, after coculture of tumour cells and T cells in 12-well plates for 7 days, each well was washed with PBS twice to remove T cells, and the surviving tumour cells were fixed and stained with crystal violet solution.

### Confocal immunofluorescence (IF) assay

IF assays were performed in both cancer cells and tumour xenograft paraffin sections. Cells were fixed with 4% paraformaldehyde for 15 min, permeabilized with 0.3% Triton X-100 for 3 min and then blocked with 10% goat serum for 30 min. Primary antibodies against Jab1 (1:200, Proteintech, Cat. No. 27511-1-AP), PD-L1 (1:300, Proteintech, Cat. No. 66248-1-Ig) were diluted in PBS and incubated at 4 °C overnight. Goat anti-rabbit Alexa 488 and anti-mouse Alexa 594 were used as secondary antibodies. The nuclear was stained with DAPI.

For tumour tissues, samples were soaked in dimethyl benzene for 15 min twice and then washed with ethyl alcohol according to a concentration gradient. Antigen retrieval was conducted with citrate antigen retrieval solution (pH = 6.0), and samples were blocked with 10% goat serum. Primary antibodies against granzyme B (1:200, Abcam, Cat. No. ab255598) were added to the samples and incubated at 4 ℃ overnight. Goat anti-rabbit Alexa 488 was used as the secondary antibody for 50 min at room temperature. The nuclear was stained with DAPI. Images were collected by confocal microscope.

#### Statistical analysis

All data were analyzed and graphed using SPSS v23.0 or GraphPad Prism 7.0. The experimental data are presented as the means ± SD. A two group comparison was analyzed by Student’s t-test, and multiple group comparison was analyzed by one-way ANOVA + two-side Dunnett test or one-way ANOVA + two-side Tukey test. The survival analysis was performed by Kaplan–Meier Curve. The correlation between α5-nAChR and PD-L1 proteins in LUAD was determined by Pearson chi-square. *P* < 0.05 was considered statistically significant.

## Results

### *CHRNA5* and *CD274* expression correlates with smoking status and poor prognosis in lung adenocarcinoma from datasets

**Fig. 1:**
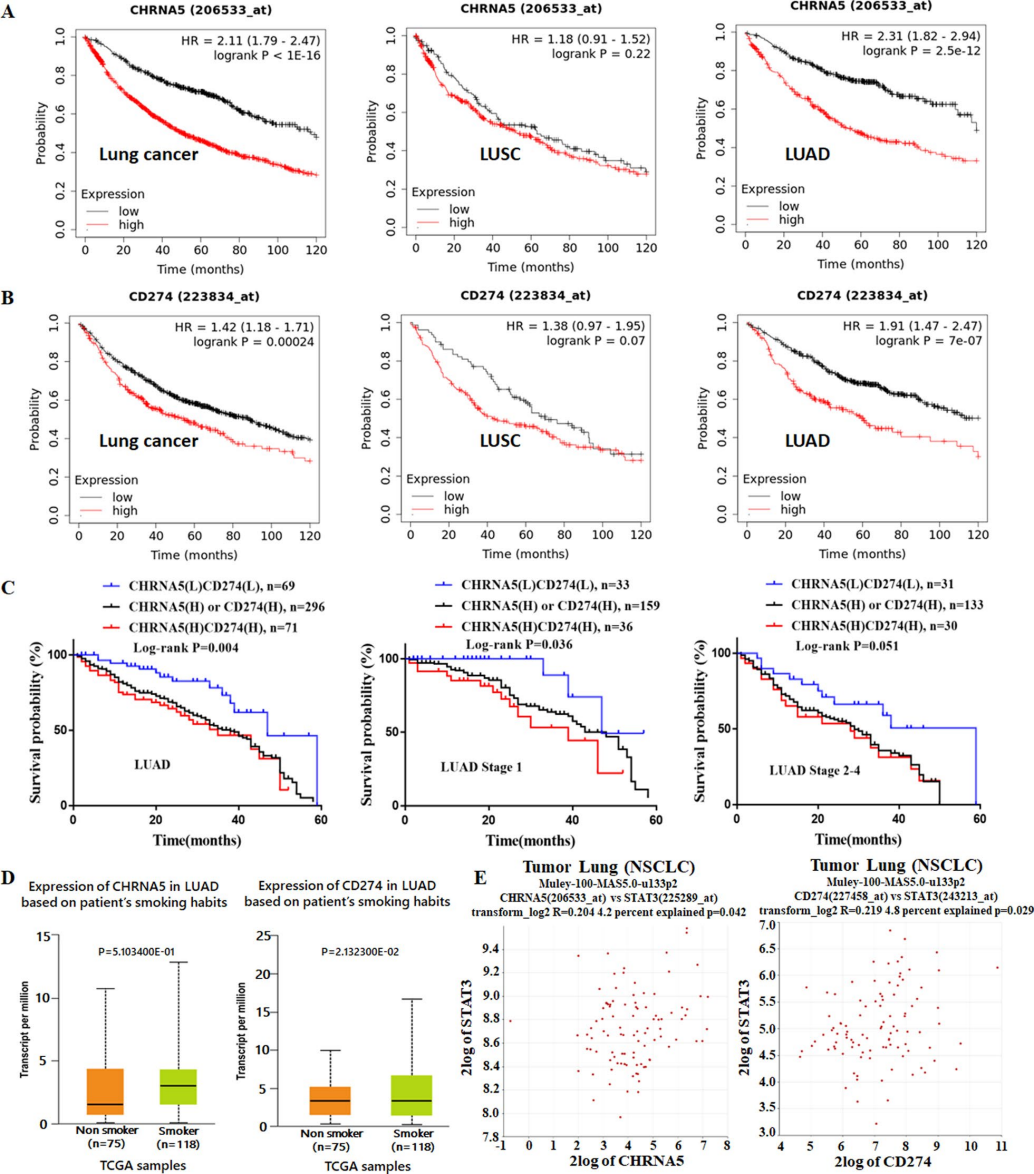
*CHRNA5* and *CD274* expression is correlated in the lung cancer dataset. **A** *CHRNA5* expression correlated with poor prognosis, especially in LUAD. Lung cancer, n = 1925. LUSC, n = 524. LUAD, n = 719. **B** *CD274* expression correlated with poor prognosis, especially in LUAD. Lung cancer, n = 1144. LUSC, n = 271. LUAD, n = 672. **C** The coexpression of *CHRNA5* and *CD274* and clinical outcomes in LUAD. LUAD, n = 365. LUAD Stage 1, n = 192. LUAD Stage 2–4, n = 164. **D** Expression of *CHRNA5* and *CD274* in LUAD smokers and non-smokers. Smokers, n = 118. Non-smoker, n = 75. **E** Correlation among the expression of *CHRNΑ5*, *STAT3* and *CD274* in LUAD

The survival analysis using the TCGA lung cancer subset from Kaplan–Meier plotter showed that high *CHRNΑ5* or *CD274* expression correlated with poor prognosis, especially in lung adenocarcinoma, but not in lung squamous cell carcinoma (LUSC) (Fig. 1A, B). To further test the prognostic value of high coexpression of *CHRNA5* and *CD274* in LUAD, we downloaded α5-nAChR and PD-L1 gene expression data from the cancer genomic browser of UCSC Xena. The survival time was lower for patients with high *CHRNA5* and *CD274* coexpression than for those with high expression of *CHRNA5* or *CD274* alone or with low coexpression (Fig. 1C, left panel). We also analyzed Kaplan–Meier survival for *CHRNA5* and *CD274* in stage 1 and stage 2–4 LUAD patients. The results showed that high *CHRNA5* and *CD274* coexpression was associated with shorter OS in early-stage patients (Fig. 1C, middle panel). Similar results were seen among stage 2–4 LUAD patients (Fig. 1C, right panel). High expression of both *CHRNA5* and *CD274* could be used as a negative index for survival in primary LUAD. *CHRNΑ5* and *CD274* expression levels were higher in LUAD smokers than in nonsmokers using the UALCAN online database (Fig. 1D). To date, there was no direct correlation between *CHRNA5* and *CD274* in LUAD, while *CHRNΑ5* and *STAT3* expression were positively correlated. Likewise, there was a positive correlation between *STAT3* and *CD274* from the R2 online database (Fig. 1E). These results suggested that *CHRNA5* and *CD274* may be correlated via *STAT3* signalling in LUAD.

### Expression of α5-nAChR correlates with PD-L1 expression and smoking status in lung adenocarcinoma specimens

**Fig. 2:**
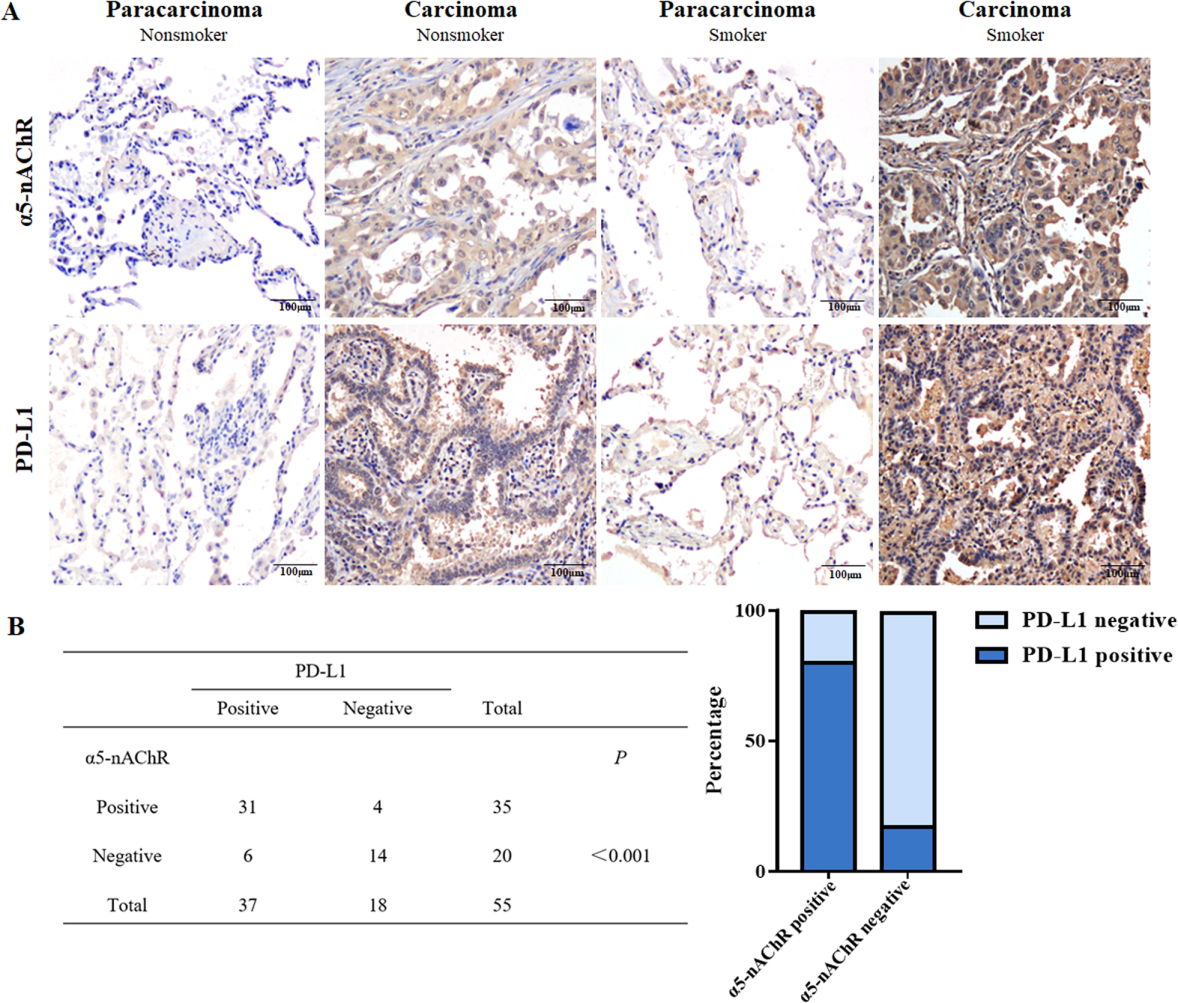
α5-nAChR and PD-L1 expression in LUAD tissue microarray. **A** α5-nAChR and PD-L1 expression in LUAD carcinoma and paracarcinoma of nonsmokers and smokers. Scare bar, 100 μm. **B** Correlation between α5-nAChR and PD-L1 expression in 55 adenocarcinoma specimens

α5-nAChR and PD-L1 expression was examined via immunohistochemistry in tissue microarrays containing 55 LUAD and 53 paracarcinoma tissue samples. The results demonstrated that α5-nAChR and PD-L1 protein levels in LUAD tissues (63.7%, 33/55; 67.3%, 37/55) were significantly higher than those in adjacent noncancerous tissues (Fig. 2A). The expression of α5-nAChR and PD-L1 in smoking samples was higher than that in nonsmoker samples. α5-nAChR expression was positively correlated with PD-L1 expression in LUAD patients (Fig. 2B). Additionally, the expression of α5-nAChR and PD-L1 was correlated with smoking status (Table 1).

**Table 1:** Correlations of α5-nAChR and PD-L1 expression with clinical parameters in lung adenocarcinoma specimens

Clinical pathology	Case (n = 55)	α5-nAChR		*P*	PD-L1		*P*
		Positive	Negative		Positive	Negative	
Sex							
Male	30	18	12	0.539	22	8	0.294
Female	25	17	8		15	10	
Age (years)							
≤ 60	19	11	8	0.520	10	9	0.093
> 60	36	24	12		27	9	
Smoking history							
No	30	15	15	0.021	16	14	0.017
Yes	25	20	5		21	4	

### α5-nAChR regulates pSTAT3, Jab1 and PD-L1 expression in LUAD cells

**Fig. 3:**
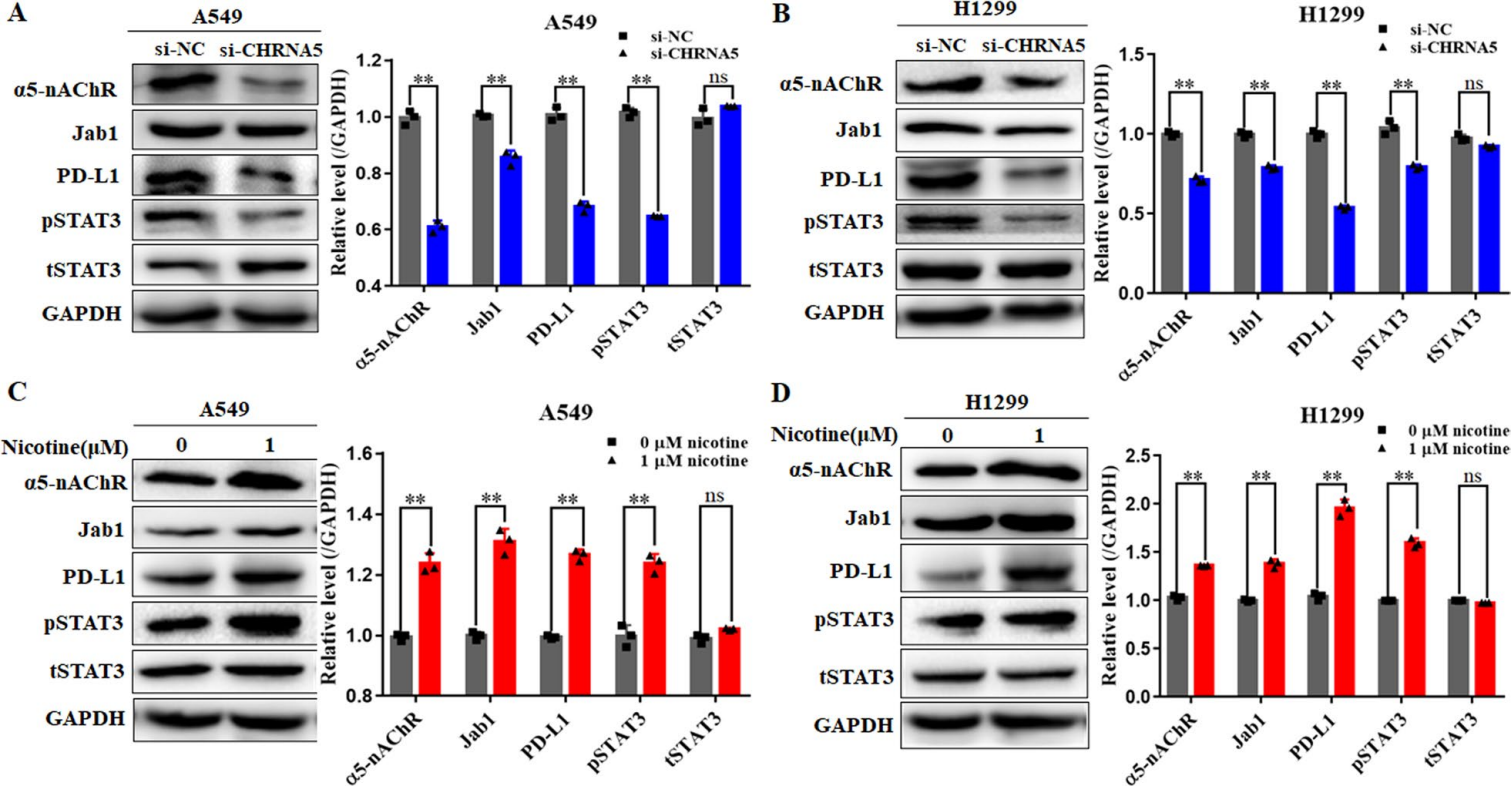
α5-nAChR mediates pSTAT3, Jab1 and PD-L1 expression in vitro. **A**, **B** Silencing α5-nAChR downregulated pSTAT3, Jab1 and PD-L1 expression in A549 cells and H1299 cells. **C**, **D** Nicotine promoted α5-nAChR, pSTAT3, Jab1 and PD-L1 expression in A549 cells and H1299 cells. Data are presented as the mean ± SEM of three independent experiments. **P < 0.01, ns not significantly different

To further understand the relationship between α5-nAChR and PD-L1 in LUAD cells, we performed western blotting to assess pSTAT3, Jab1 and PD-L1 protein levels in α5-nAChR-knockdown and nicotine-treated cells. The results showed that the protein levels of pSTAT3, Jab1 and PD-L1 were downregulated in the CHRNA5-siRNA-expressing A549 and H1299 cells compared to the scrambled siRNA expressing A549 and H1299 cells (Fig. 3A, B). On the other hand, substantially, the protein levels of pSTAT3, Jab1 and PD-L1 were upregulated in A549 and H1299 cells (Fig. 3C, D) treated with 1 μM nicotine for 16 h. These results are consistent with those of our former study showing that α5-nAChR/STAT3/Jab1 signalling contributed to lung carcinogenesis [26] and suggested that α5-nAChR mediates PD-L1 expression in NSCLC cells.

### α5-nAChR mediates PD-L1 expression via STAT3 binding to PD-L1 or Jab1 promoter in NSCLC cells

**Fig. 4:**
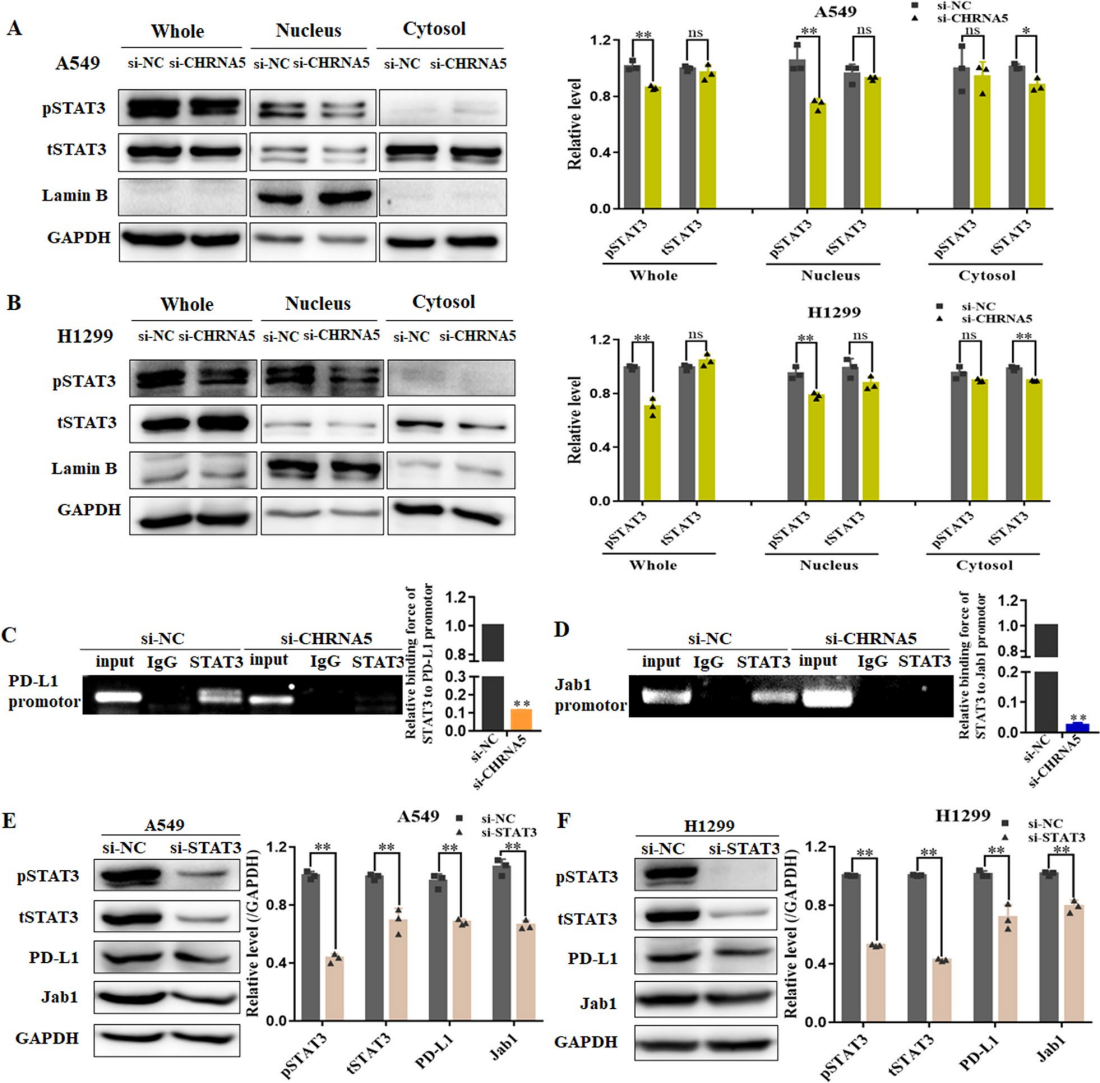
α5-nAChR mediates PD-L1 expression via STAT3 binding to the PD-L1 or Jab1 promoter in LUAD cells. **A**, **B** pSTAT3 expression in the whole, nuclear, and cytoplasmic lysates of A549 cells and H1299 cells. **C**, **D** STAT3 bound to the PD-L1 and Jab1 promoter in A549 cells. **E**, **F** Silencing STAT3 downregulated PD-L1 and Jab1 expression in Α549 cells and H1299 cells. Data are presented as the mean ± SEM of three independent experiments. *P < 0.05, **P < 0.01, ns not significantly different

We further confirmed that α5-nAChR could regulate the translocation of pSTAT3 into the nucleus for PD-L1 or Jab1 expression. Notably, nuclear pSTAT3 was lower in cells expressing si-CHRNA5 than in those expressing si-NC (Fig. 4A, B). Recent findings have mentioned that fragments of the PD-L1 or Jab1 core promoter are enriched with consensus binding sites for STAT3, which are known to regulate PD-L1 or Jab1 expression transcriptionally [27, 28]. We performed a ChIP assay with an anti-STAT3 rabbit polyclonal antibody and primer pairs specific for the PD-L1 gene promoter in the si-CHRNA5 or si-NC group of A549 cells. The results showed that the PD-L1 level was decreased by STAT3 binding to the PD-L1 promoter in the si-CHRNA5 group compared to the si-NC group (Fig. 4C). Meanwhile, to investigate whether α5-nAChR influenced PD-L1 expression via STAT3-Jab1 signalling in NSCLC, we performed a ChIP assay to identify STAT3 binding to the Jab1 promotor. Knockdown of α5-nAChR significantly decreased the amount of STAT3 binding to the Jab1 promoter in A549 cells (Fig. 4D). Moreover, the protein levels of PD-L1 and Jab1 were downregulated in A549 and H1299 cells expressing STAT3-siRNA compared to those expressing scrambled siRNA (Fig. 4E, F). These results suggested that α5-nAChR mediates PD-L1 expression via STAT3 binding to the PD-L1 or Jab1 promoter in NSCLC cells.

### Jab1 stabilizes PD-L1 expression in NSCLC cells

**Fig. 5:**
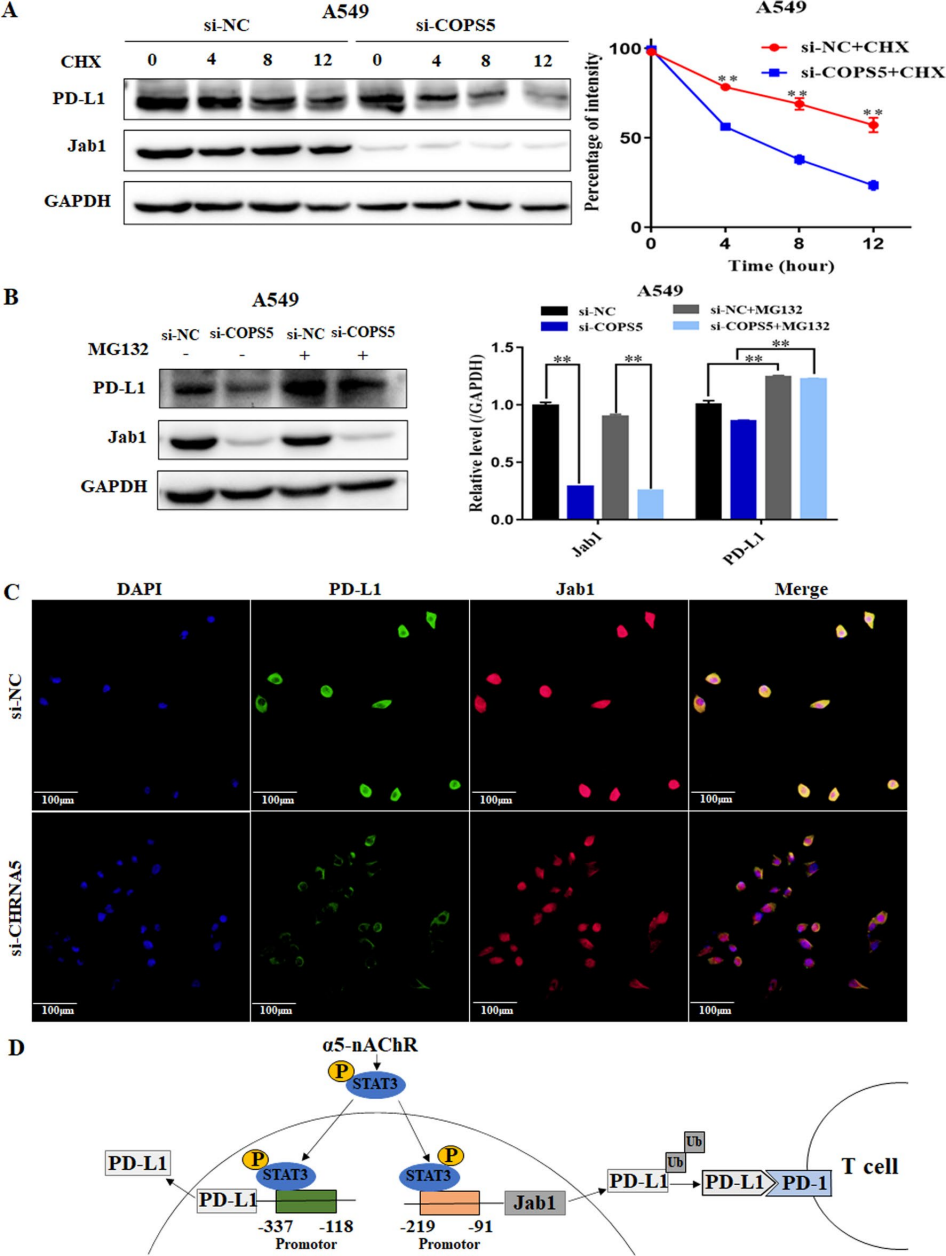
Jab1 stabilizes PD-L1 expression in LUAD cells. **A** PD-L1 expression in the si-NC or si-COPS5 groups treated with 50 μg/ml CHX. **B** PD-L1 expression in the si-NC or si-COPS5 groups treated with 10 μM MG-132 for 24 h. **C** Immunofluorescence staining of PD-L1 (green) and Jab1 (red). Scale bar, 100 μm. **D** The STAT3 binding site of the human Jab1 and PD-L1 promoter. Data are presented as the mean ± SEM of three independent experiments. **P < 0.01

Studies has shown that Jab1 mediates PD-L1 stabilization via the ubiquitination and degradation of PD-L1 [24, 29, 30]. To verify whether Jab1 affects the stability of PD-L1 in NSCLC cells, we used CHX as a protein synthesis inhibitor to inhibit the production of protein, and subsequently detected PD-L1 expression at diverse time points. PD-L1 was rapidly degraded in Jab1-siRNA A549 cells (Fig. 5A). Furthermore, Jab1-mediated downregulation of PD-L1 was inhibited by MG132, a 26S proteasome inhibitor, in A549 cells (Fig. 5B). These data indicated that Jab1 stabilizes PD-L1 expression, which was supported by colocalization of Jab1 and PD-L1 in A549 cells via double immunofluorescence staining (Fig. 5C). Our results suggested that α5-nAChR upregulates STAT3 or STAT3-Jab1 to stabilize PD-L1, thereby enhancing its interaction with PD-1 to escape T cell immune surveillance (Fig. 5D).

### α5-nAChR expression mediated an immunosuppressive effect in the cocultured system

**Fig. 6:**
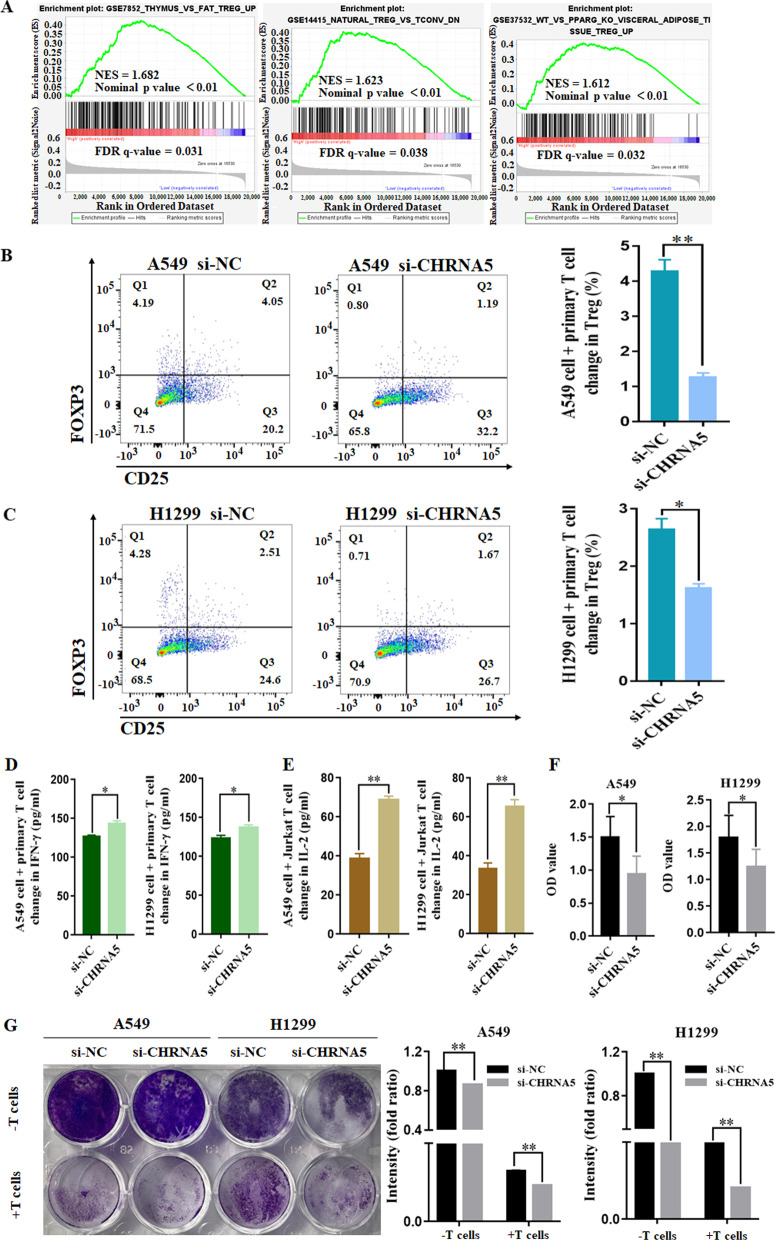
α5-nAChR mediates T cell activity in the coculture system. **A** *CHRNA5* expression was positively correlated with Tregs. **B**, **C** Tregs in T lymphocytes cocultured with A549 and H1299 cells. **D**, **E** IFN-γ and IL-2 levels in the coculture supernatant. **F** T cell-mediated cell killing by CCK-8 assay. **G** T cell-regulated tumour cell killing by crystal violet staining. Data are presented as the mean ± SEM of three independent experiments. *P < 0.05, **P < 0.01

To investigate whether the mechanism of α5-nAChR expression mediates T cell activity, we performed gene set enrichment analysis (GSEA) of TCGA data. Compared with the *CHRNA5* low expression group, the leading enriched signatures in the *CHRNA5* high expression group were positively correlated with Tregs (Fig. 6A). After primary T cells and Jurkat T cells were cocultured with A549 or H1299 LUAD cells, we evaluated the relative frequencies of CD4^+^CD25^+^FOXP3^+^ Tregs and the concentration of IFN-γ and IL-2. The frequencies of CD4^+^CD25^+^FOXP3^+^ Tregs were significantly decreased in the si-CHRNA5 group compared to the si-NC group (Fig. 6B, C). Moreover, ELISA results showed that the secretion of IFN-γ and IL-2 were increased in the si-CHRNA5 group compared to the si-NC group (Fig. 6D, E).

To confirm the role of α5-nAChR-mediated suppression of cytotoxic T-cell activity, A549 and H1299 cells were cocultured with activated T cells. CCK8 and crystal violet staining assays showed that cell proliferation obviously decreased in the si-CHRNA5 group compared to the si-NC group in A549 and H1299 cells (Fig. 6F, G), which suggested that T cell-mediated killing was increased after silencing α5-nAChR. Collectively, these data indicated that α5-nAChR inhibits cytotoxic T-cell activity by regulating PD-L1 expression in vitro.

### α5-nAChR expression was related to PD-L1, Jab1, pSTAT3 and GB expression in LUAD xenografts tissues of BALB/c nude mice

**Fig. 7:**
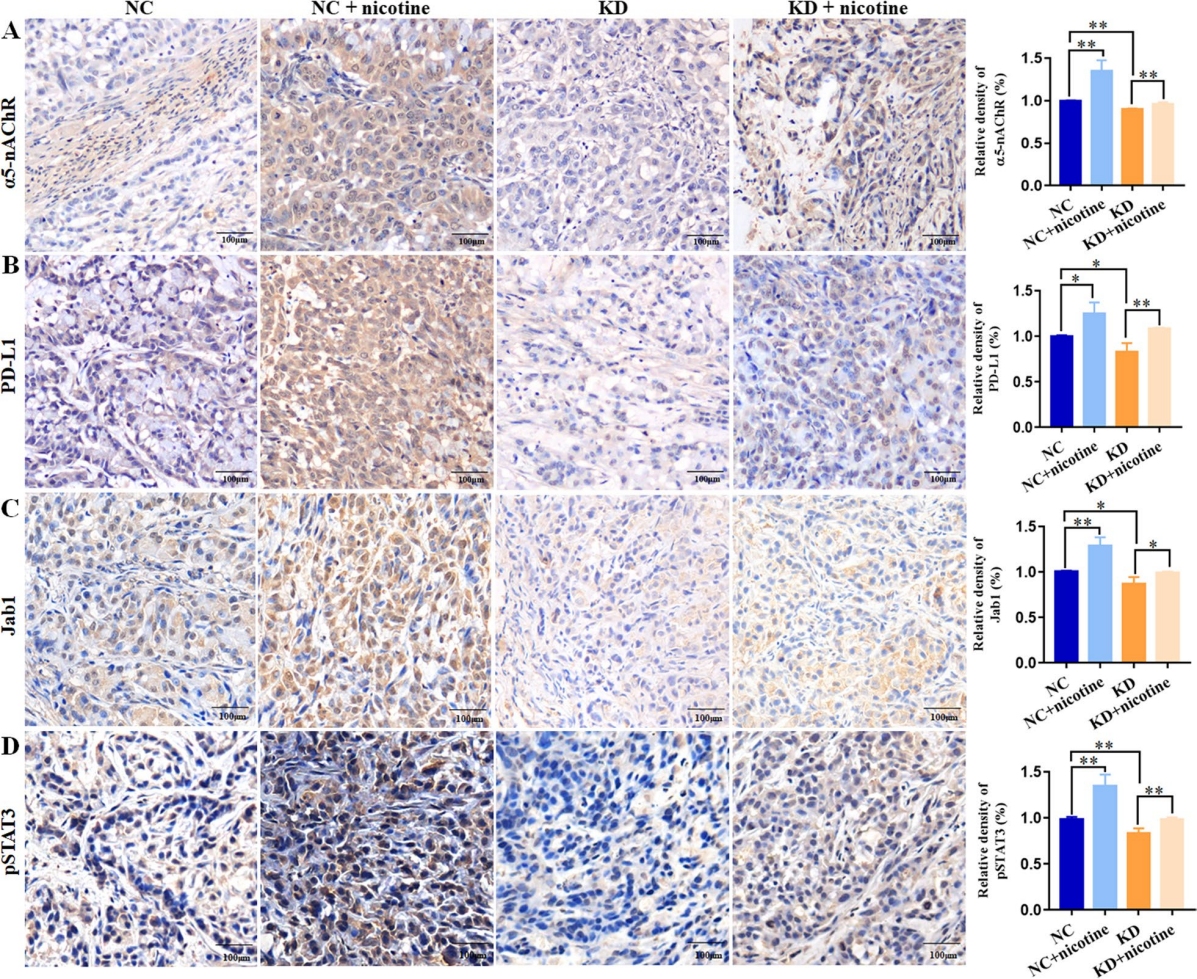
α5-nAChR, PD-L1, Jab1 and pSTAT3 expression in LUAD tumour xenograft tissues of nude mice. **A** α5-nAChR expression in NC, NC + nicotine, KD and KD + nicotine groups. **B** PD-L1 expression in NC, NC + nicotine, KD and KD + nicotine groups. **C** Jab1 expression in NC, NC + nicotine, KD and KD + nicotine groups. **D** pSTAT3 expression in NC, NC + nicotine, KD and KD + nicotine groups. Scare bar, 100 μm. Data are presented as the mean ± SEM of three independent experiments. *P < 0.05, **P < 0.01

As α5-nAChR expression was observed to be associated with PD-L1, Jab1, pSTAT3 expression in vitro, we further assessed α5-nAChR, PD-L1, Jab1, pSTAT3 expression in LUAD xenograft tissues. The expression of α5-nAChR (Fig. 7A), PD-L1 (Fig. 7B), Jab1 (Fig. 7C), pSTAT3 (Fig. 7D) was higher in the NC group than in the KD group. In addition, in nicotine-treated xenograft tissues, α5-nAChR, PD-L1, Jab1, pSTAT3 expression was higher than that in the NC group tissues. This result was also verified in the KD group and the nicotine-treated KD group. These results suggest that α5-nAChR is positively associated with PD-L1, Jab1, pSTAT3 expression in LUAD xenograft tissues.

**Fig. 8:**
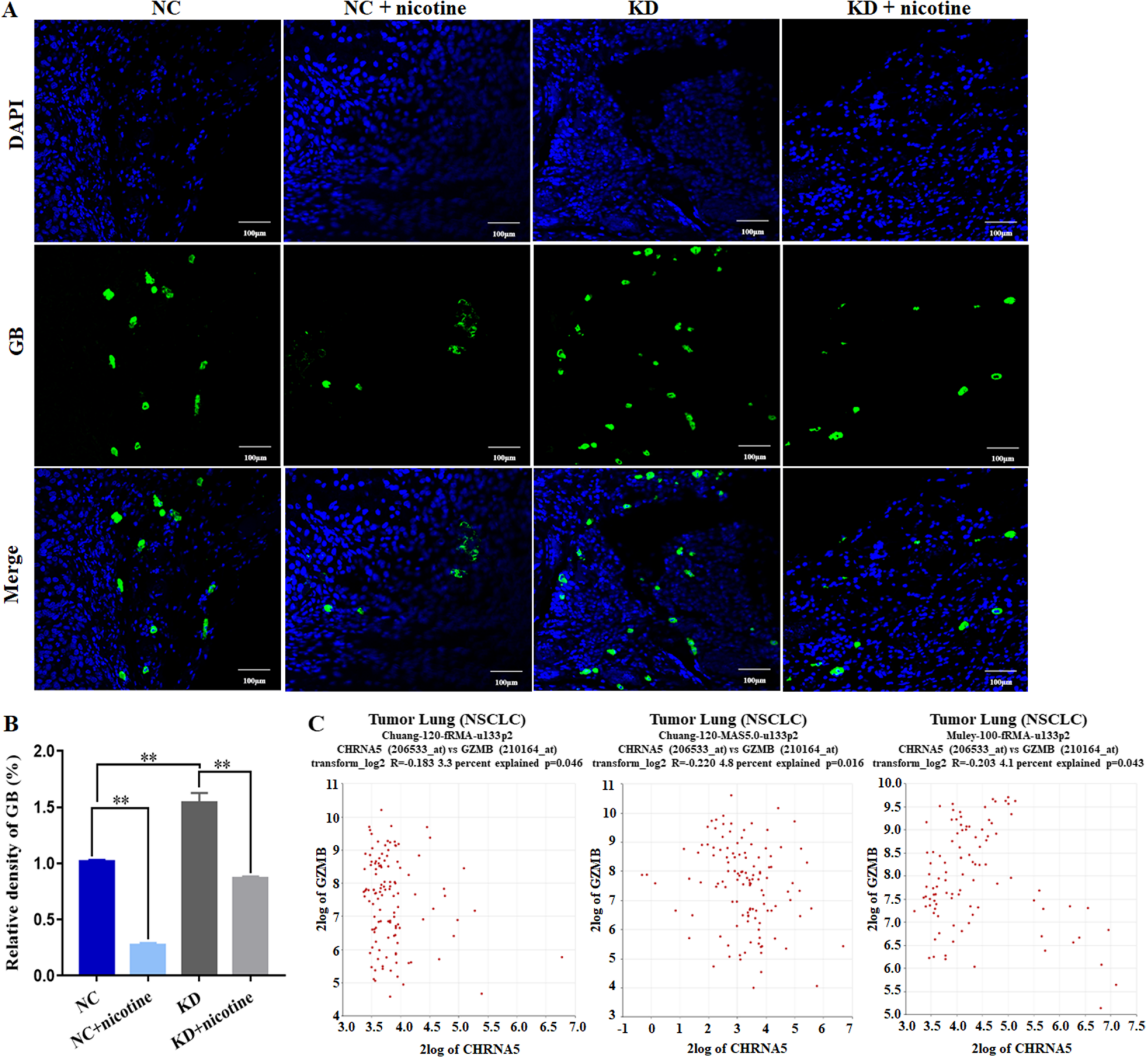
Knockdown of α5-nAChR promotes granzyme B secretion in LUAD tumour xenografts. **A** Granzyme B in mouse tumour tissues of each group. Scare bar, 100 μm. **B** The relative density of GB. **C** Correlation between the expression of *CHRNΑ5* and *GZMB*. Data are presented as the mean ± SEM of three independent experiments. **P < 0.01

Previous studies have shown that CD8^+^ T cells and NK cells can recognize and remove tumour cells by secreting granzyme B [31]. Therefore, we detected the expression of granzyme B in LUAD tumour xenografts by IF assay. The results showed that α5-nAChR knockdown promoted granzyme B secretion in tumour tissues. Granzyme B was lower in nicotine-treated xenograft tissues than in the NC group tissues. Meanwhile, granzyme B was higher in the KD group than in the nicotine‐treated KD group (Fig. 8A, B). We further demonstrated that there was a negative correlation between *CHRNA5* and *GZMB* from the R2 online database (Fig. 8C). This result suggested that *CHRNA5* and *GZMB* may be correlated in LUAD.

### α5-nAChR mediated T cell activity via PD-L1 in C57BL/6 mice model

**Fig. 9:**
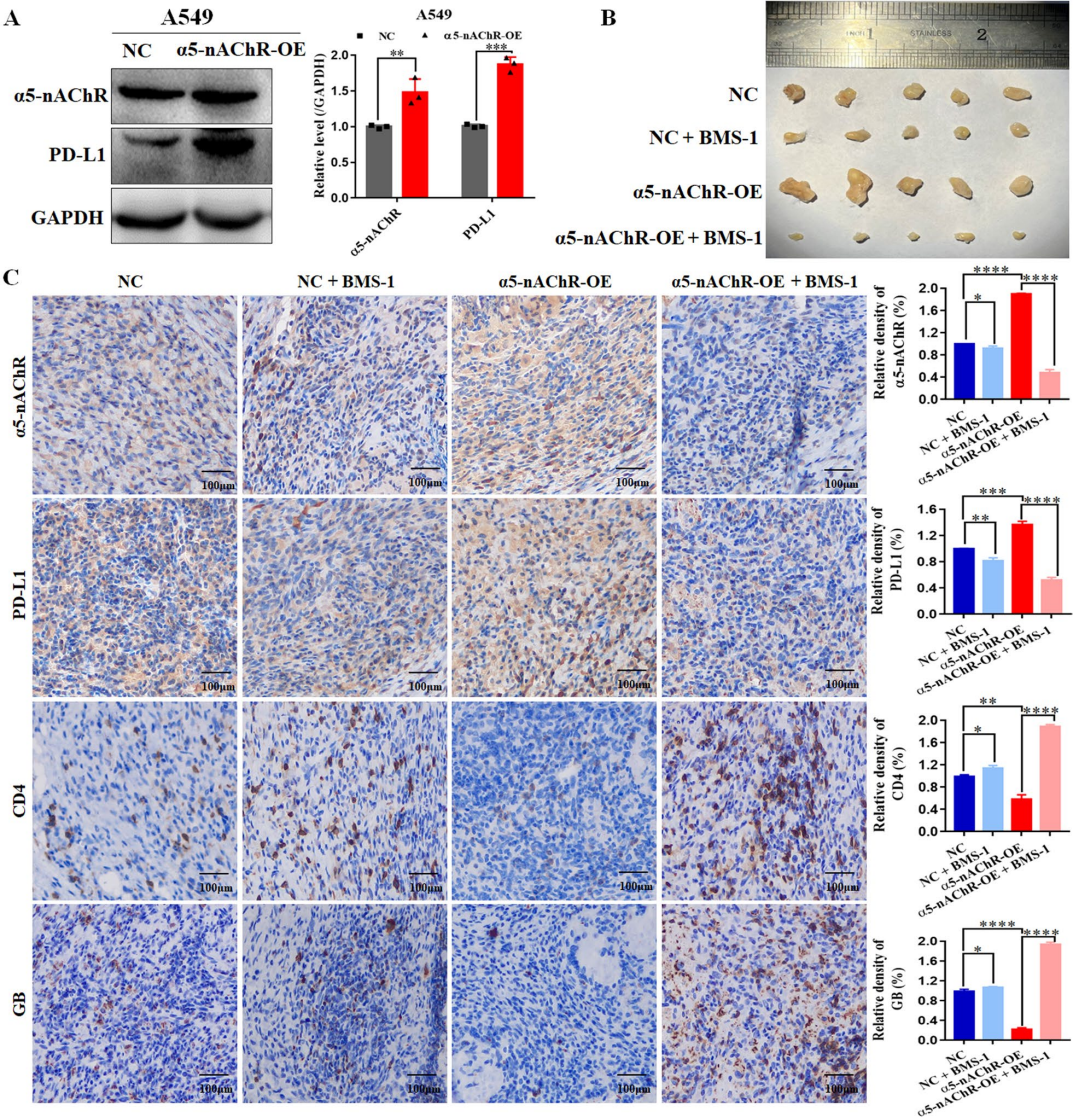
α5-nAChR promotes tumour growth and immune escape by upregulating PD-L1 in C57BL/6 mice model. **A** Stable α5-nAChR overexpression upregulated PD-L1 expression. **B** The morphology of tumour xenografts from each mouse was photographed. **C** The expression level of PD-L1, CD4 and GB within tumour xenografts in each group of C57BL/6 mice, N = 5 mice/group. Scare bar, 100 μm. *P < 0.05, **P < 0.01, ***P < 0.001, ****P < 0.0001

To further verify the regulation of α5-nAChR/PD-L1 signalling on lung cancer growth and immune escape, we implanted the human A549 cell lines into C57BL/6 mice. We established the A549 cell lines with stable α5-nAChR overexpression (Fig. 9A). The results in vivo showed that overexpression of α5-nAChR significantly promoted tumor growth, whereas this effect was reduced after BMS-1 treatment (Fig. 9B). Moreover, the immunohistochemical analysis of tumor tissues similarly revealed that α5-nAChR overexpression mediated the expression of PD-L1, CD4 and GB expression (Fig. 9C). These data confirmed that α5-nAChR-mediated tumor growth and immune escape was realized through the PD-L1 signalling in lung cancer.

## Discussion

Tobacco smoking remains the leading preventable risk factor for cancer, yet it accounts for more than 30% of total cancer deaths and over 80% of lung cancer cases [32]. Increasing evidence indicates that nicotine is one of the harshest substances in tobacco smoke, contributes to carcinogenesis in various cancer types, affects cancer progression and results in a poor prognosis in patients. Nicotine binds to nAChRs and induces various downstream signalling pathways involved in cancer promotion, progression, and malignancy [33]. Investigators have demonstrated that overexpression of α7-nAChR induces cholangiocarcinoma progression by blocking apoptosis and promoting the EMT process [34]. Parasympathetic nerves may promote the progression of CRC through α9-nAChR [35]. A common polymorphism, rs16969968, encoded in the α5 nicotinic acetylcholine receptor subunit gene, is a well-recognized marker for smoking risk [36, 37]. Our previous study demonstrated that nicotine interacts with α5-nAChR, activating the JAK2/STAT3 and Jab1 signalling pathways and contributing to lung carcinogenesis [19, 20, 26, 38]. These data indicate that α5-nAChR plays an important role in lung carcinogenesis.

The introduction of the immune checkpoint inhibitors (ICIs) in clinical practice radically changed the treatment algorithm of lung cancers [39]. Anti-PD-1 agents are now standard in the treatment of advanced NSCLC, either as monotherapy in those with PD-L1 tumour expression or in combination with chemotherapy in all patients [40]. Inhibition of PD-1 and PD-L1 interactions can lead to restored T-cell function and antitumour activity. A previous study demonstrated that an increased smoking history is an important factor in predicting the response to ICIs in patients with advanced NSCLC [41]. Cigarette smoking is generally associated with T-cell exhaustion, and upregulation of PD-L1 leads to immune evasion [42, 43]. The benefits of being a smoker hint at the existence of PD-1/PD-L1 sensitizers for patients on single-agent immunotherapy [44]. The above results suggest the possibility that nicotine may induce a mechanism that drives the expression of PD-L1 in NSCLC cells via α5-nAChR.

The transcription factor STAT3 plays a key role in the regulation of PD-L1 expression in various cancer types [45]. In non-Hodgkin lymphoma, STAT3 binds to the PD-L1 gene promoter, which upregulates PD-L1 expression in vitro and in vivo [46]. Dandelion extract inhibited the proliferation, migration and invasion of TNBC cells in the TAM microenvironment by suppressing the IL-10/STAT3/PD-L1 immunosuppressive signalling pathway [47]. Shikonin inhibited immune evasion in PC by inhibiting PD-L1 glycosylation and activating the NF-κB/STAT3 and NF-κB/Jab1 signalling pathways [25]. Jab1 is a key regulatory factor involved in various tumorigenesis-associated signalling pathways and is a potential target for smoking-induced lung cancer [22]. Moreover, Jab1 is required for PD-L1 stabilization in cancer cells and promotes tumour progression and migration [27]. In our previous study, α5-nAChR mediated LUAD cells EMT, migration and invasion via STAT3/Jab1 in LUAD [26]. In this study, the results showed that the expression of α5-nAChR was correlated with PD-L1 expression, smoking status and lower survival of NSCLC in vivo. PD-L1 levels were decreased by STAT3 binding to the PD-L1 or Jab1 promoter in the si-CHRNA5 group compared to the si-NC group. Moreover, Tregs and cytotoxic T cells activities were mediated in the si-CHRNA5 group compared to the si-NC group by cocultured assay. These results suggest that the α5-nAChR-STAT3-Jab1 axis mediates immune escape by regulating PD-L1 expression in lung cancer.

## Conclusions

**Fig. 10:**
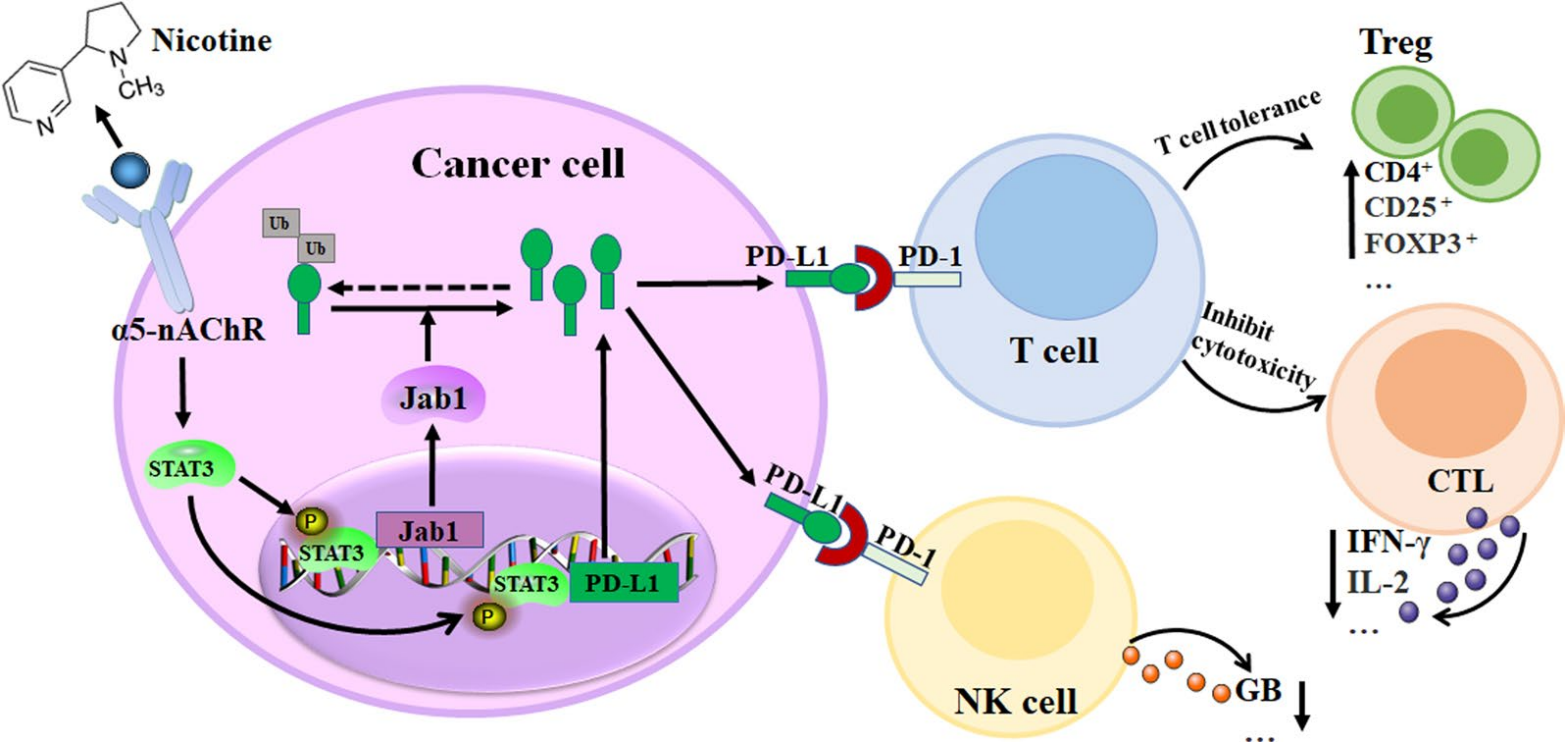
Proposed signalling cascades of α5-nAChR and PD-L1 involves in immune escape in lung cancer. Nicotine binds and interacts with α5-nAChR on the cell surface, activates STAT3/PD-L1 or STAT3/Jab1-PD-L1 signalling, thereby α5-nAChR/PD-L1 axis mediated immune cells activity in lung carcinogenesis

In conclusion, α5-nAChR expression is positively associated with PD-L1. These findings provide new insights into the possible molecular mechanisms of α5-nAChR underlying PD-L1-mediated immune escape in lung cancer. Acetylcholine and its analogue bind and interact with α5-nAChR on the cell surface and activate STAT3/PD-L1 or STAT3/Jab1-PD-L1 signalling, which subsequently mediates immune cell activity in lung carcinogenesis (Fig. 10). To the best of our knowledge, this is the first study to demonstrate that α5-nAChR is involved in immune escape via STAT3/PD-L1 or STAT3/Jab1-PD-L1 signalling in lung cancer, which may be useful as a potential target for NSCLC diagnosis and immunotherapy in the future.

## Data Availability

The authors confirm the data that has been used in this work is available on reasonable request.

## Abbreviations

NSCLC: Non-small-cell lung cancer
PD-L1: Programmed cell death ligand-1
GWAS: Genome-wide association studies
α5-nAChR: Alpha5-nicotinic acetylcholine receptor
EMT: Epithelial-mesenchymal transition
Tregs: Regulatory T cells
pSTAT3: Phosphorylated STAT3
NK: Natural killer cell
EGFR: Epidermal growth factor receptor
ALK: Anaplastic lymphoma kinase
PD-1: Programmed cell death protein 1
OS: Overall survival
PBMCs: Peripheral blood mononuclear cells
